# Pyrraline Formation Modulated by Sodium Chloride and Controlled by Encapsulation with Different Coating Materials in the Maillard Reaction

**DOI:** 10.3390/biom9110721

**Published:** 2019-11-10

**Authors:** Zhili Liang, Xu Chen, Zhao Yang, Yuzhu Lai, Yinling Yang, Chuying Lei, Ya Zeng

**Affiliations:** 1 School of Food Science, Guangdong Food and Drug Vocational College, Guangzhou 510520, China; yangzhao19878@163.com (Z.Y.); laiyuzhuhu@icloud.com (Y.L.); yang114545@163.com (Y.Y.); Lchuying1029@163.com (C.L.); Alina112697@163.com (Y.Z.); 2 Engineering Research Center of Health Food Design & Nutrition Regulation, School of Chemical Engineering and Energy Technology, Dongguan University of Technology, Dongguan 523808, China; chenxu@dgut.edu.cn

**Keywords:** encapsulation, sodium chloride, advanced glycation end products, starch, gum

## Abstract

Advanced glycation end products (AGEs), which are present in heat-processed foods, have been associated with several chronic diseases. Sodium chloride (NaCl) modulates the formation of furfurals and acrylamide in the Maillard reaction; however, the effects of NaCl on AGE formation are inconsistent. In this study, we investigated the effects of NaCl on pyrraline formation using glucose-lysine model systems. NaCl, especially at 0.50%, promoted Maillard browning and pyrraline formation, with a simultaneous increase in the 3-deoxyglucosone concentration. To reduce the rate of pyrraline formation, NaCl coated with different gums and starches were used. The results showed that NaCl encapsulation is an effective approach to mitigate pyrraline and 3-deoxyglucosone formation. The content of NaCl in the microparticles were 284 ± 12, 269 ± 6, 258 ± 8, 247 ± 10, 273 ± 16, and 288 ± 15 mg/g (coated with waxy maize starch, normal maize starch, HYLON VII high amylose maize starch, gelatinized resistant starch, xanthan gum, and gum arabic, respectively). The heat resistance of the coating material was negatively correlated with the pyrraline and 3-deoxyglucosone formation, whereas the solubility of the coating material had the opposite results. Coating the material with gum had little effects on the reduction of pyrraline and 3-deoxyglucosone.

## 1. Introduction

A non-enzymatic browning reaction (i.e., the Maillard reaction (MR)) in heat-processed foods begins between an amino acid and a reducing sugar, and is followed by a cascade of reactions. These reactions produce different intermediates, including aroma compounds and high molecular weight brown polymers [1]. In the food industry, desirable colors and aromas of foods (e.g., bread, meat, roasted nut, coffee, and confectionaries) are generated by MR during food processing. However, some compounds produced during MR are potentially harmful, including acrylamide, heterocyclic amines (HCAs), and advanced glycation end products (AGEs).

AGEs are modified-structure compounds produced in the late stage of MR. Dietary AGEs (d-AGEs) formation are rapidly stimulated by the heating temperature and time. AGEs have been associated with several chronic diseases, including diabetes [2,3] and kidney disorders [4,5,6]. In patients with diabetes, renal failure, overweight, or obesity, d-AGEs can modulate the AGE load in the body [7,8,9]. A high intake of d-AGEs may increase the risk of chronic diseases [10,11]; the restriction of d-AGEs can alleviate the burden of health risks associated with AGEs [12,13]. D-AGEs are generally associated with the glycation of lysine and arginine; however, lysine-derived AGEs, such as N^ε^-carboxymethyllysine (CML), N^ε^- carboxyethyllysine (CEL), and pyrraline, are generally used as d-AGE markers [14,15,16].

**Scheme 1:**
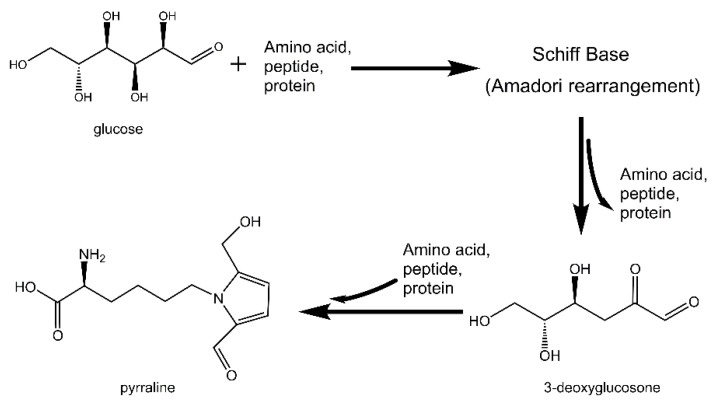
The proposed pathway of pyrraline formation.

Pyrraline is one of the common AGEs, which has been be found predominantly in various food products after a high thermal impact. For example, up to134 mg/kg protein pyrraline can be found in in processed carrot juice [17]. In bread crusts or rusks, the content of pyrraline can even range up to 3680 mg/kg protein [18]. 3-deoxyglucosone (3-DG), one of the major dicarbonyl compounds in the Maillard reaction, is a key intermediate in the pathway of pyrralline formation. As shown in Scheme 1, pyrraline is formed by the addition of 3-DG to the ε-amino group of lysine residues on proteins [19].

In the food industry, the reduction and/or prevention of d-AGEs formation is of the utmost importance. Monovalent, bivalent, and polyvalent cations, such as Na^+^ and Ca^2+^, may affect MR through the dehydration of various key intermediates. For example, the addition of Ca^2+^ prevents acrylamide formation in an asparagine–glucose model system, but simultaneously boosts the formation of hydroxymethylfurfural (HMF) [20]. Levine et al. [21] reported that calcium chloride (CaCl_2_) and sodium chloride (NaCl) reduced acrylamide by 36% and 23%, respectively, in a dough model system. However, Claus et al. [22] concluded that NaCl plays an inconsistent role in acrylamide formation—while the acrylamide concentration declined at 1–2% NaCl, >2% NaCl increased acrylamide concentration. Similar results were obtained in an asparagine–glucose model system—acrylamide concentration declined with 0.5–5 μmol/L NaCl and increased with 5–20 μmol/L NaCl [20]. To prevent the HMF and acrylamide formation in food, researchers have evaluated the NaCl encapsulation. Fiore et al. [23] reported that lipid-coated NaCl can significantly prevent HMF formation in thermally processed foods. Therefore, in the food industry, the addition of NaCl can not only improve the sensory properties of foods by increasing the saltiness, texture, and other congruent flavor effects [24], but can also impact on the furfurals and acrylamide formations.

Even though there is considerable knowledge on the effect of NaCl on the furfurals and acrylamide formations during the heating of foods, there is little information on the effects of NaCl on d-AGE formation. We hypothesize that the NaCl concentration affects the AGE formation in MR. In this study, we evaluated the effect of NaCl on pyrraline formation using an established glucose–lysine model system. Additionally, we used NaCl coated with different gums and starches, and monitored the formation of pyrrraline and 3-DG at different heating temperatures and times.

## 2. Materials and Methods

In conventional cooking, the temperatures of the thermal-induced MR range between 100 and 300 °C [25]. Certain AGEs, such as CML and pyrraline, are likely to form at 120–200 °C [26,27,28]. Some studies [29,30,31,32,33] have reported that the sodium content (expressed as a percentage of mass concentration) ranges between 0.00% and 1.00% in foods, including packaged, commercially processed, and restaurant foods. Therefore, in this study, we used a temperature range of 140–180 °C and a sodium content of 0.00–1.00%.

### 2.1. Chemicals and Reagents

All of the chemicals were of analytical grade, unless otherwise stated. The L-lysine, D-glucose, NaCl, xanthan gum, and gum arabic were purchased from Macklin Biochemical Co., Ltd. (Shanghai, China). The acetonitrile and formic acid of HPLC grade were acquired from Merck (Darmstadt, Germany). A solid-phase extraction cartridge, Cleanert PEP-2 (200 mg/6 mL, Bonna-Agela Technologies Inc., Tianjin, China), was used for the purification of the MR products. The pyrraline (purity >99.99%) and 3-deoxyglucosone (purity >99.99%) were supplied by Toronto Research Chemicals (North York, Ontario, Canada). The starches were obtained from Ingredion Incorporated (Shanghai, China).

### 2.2. Preparation of Glucose–Lysine–NaCl Model Systems

A model system consisting of lysine, glucose, and NaCl was used to determine the effect of NaCl on pyrraline formation. Lysine (0.1 mmol), glucose (0.1 mmol), and NaCl (set concentration) were transferred to a 25-mL polytetrafluoroethylene (PTFE)-lined hydrothermal autoclave reactor (Tefic Biotech Co., Xi’an, China). The total reaction volume in the PTFE-lined tube was adjusted to 15 mL, using deionized water. The final concentrations of Na^+^ (not NaCl) in the reaction solution were 0.00%, 0.25%, 0.50%, 0.75%, and 1.00%. The concentration of Na^+^ was 39.3% of the NaCl concentration. The tube was covered with a PTFE lid and inserted into the stainless-steel jacket, and the jacket was tightly sealed with a stainless-steel cap prior to the thermal treatments. The thermal treatments were performed in an oil bath at 140, 160, and 180 °C, for 5 to 20 min. All of the reactions were performed in triplicate.

Following the thermal treatments, the autoclave reactors were immediately cooled to room temperature in an ice bath. Subsequently, 0.1 mL of a 5 mol/L ο-phenylenediamine (OPD) solution (dissolved in 1:1 methanol–water solution) was added to terminate the reaction, resulting in the derivatization of dicarbonyl compounds into azines (3-DG quinoxaline, abbreviated as 3-DG_qx_), and shaken in the dark overnight. The mixture was passed through a 0.45-μm membrane filter and stored at −18 °C prior to the solid-phase extraction (SPE) and chromatographic analysis.

### 2.3. Encapsulation Process

Microencapsulation by spray-coating was performed using an YC-015 laboratory spray dryer (Shanghai Pilotech Instrument and Equipment Co., Shanghai, China). The NaCl was coated with six different materials, namely: xanthan gum (XG), gum arabic (GA), waxy maize starch (WMS), normal maize starch (NMS), HYLON VII high amylose maize starch (HAMS), and gelatinized resistant starch (GRS).

The sprayed liquid was prepared using a coating material, NaCl, and deionized water. The NaCl (1 g) and coating material (10 g) were mixed, added to 1 L deionized water, and heated to gelatinization under constant stirring. A two-fluid nozzle with a cap orifice diameter of 0.7 mm was used. The processing conditions consisted of a drying air inlet temperature of 200 °C, a drying air outlet temperature of 80 °C, a liquid feed volumetric flow rate of 15 mL/min, an air volumetric flow pressure of 0.25 MPa, and a drying air volumetric flow rate of 60 L/h. After the completion of the experiment and when the air inlet temperature was <60 °C, the samples were collected from the product collection vessel.

### 2.4. Thermal Properties of NaCl Microparticles

The melting points of the NaCl microparticles were measured using a differential scanning calorimeter (DSC; DSC 3, Mettler Toledo, Zurich, Switzerland), previously calibrated with indium (mp = 156.6 °C, *ΔH* = 28.5 J/g). The analysis was performed in duplicate in a nitrogen gas atmosphere (30 mL/min), using an empty pan as reference. The samples (~20 mg; dry starch or gum basis) in deionized water (3×, *w*/*w*; dry starch or gum basis) were scanned from 30 °C to 180 °C at 10 °C/min in a sealed aluminum pan. The onset temperature (*T_o_*), peak temperature (*T_p_*), conclusion temperature (*T_c_*), and enthalpy change (*ΔH*) were determined. Following the first scan, the sealed aluminum pan was placed at 4 °C for 7 d for the retrogradation analysis.

### 2.5. Physical Analysis of the Microparticles

#### 2.5.1. Particle Size Distribution

The microparticle size distributions were determined in triplicate by laser granulometry (Malvern Mastersizer 2000, Malvern, United Kingdom).

#### 2.5.2. Scanning Electron Microscopy (SEM)

The microparticles were mounted on aluminum stubs with double-sided sticky carbon tape, and sputter-coated with a fine layer of gold (metallization step). The pressure was set to 10^−3^ mPa during the metallization step, and to 6 × 10^−6^ mPa inside the apparatus. The morphology of the microparticles was examined using a Zeiss EVO18 SEM instrument (Carl Zeiss, Oberkochen, Germany) operated at a 5 KV accelerating voltage.

#### 2.5.3. Content of NaCl in Microparticles

An inductively coupled plasma-optical emission spectrometer (ICP-OES, model Optima 7000 DV, PerkinElmer Inc., Boston, MA, USA) with a radial plasma configuration was used to determine the content of the sodium ions. Standard plasma conditions were used (i.e., 1300 W for radio-frequency power; 1.5 mL/min pump rate; and 15.0, 0.2, and 0.8 L/min for the plasma, auxiliary, and nebulizer gas flow, respectively). The detection wavelength for sodium was 589.592 nm.

The microparticles (0.2 g) were transferred to a 25-mL PTFE tube and mixed with 10 mL of 65% nitric acid. The mixture was shaken and covered with a PTFE lid prior to overnight storage inside a fume hood. The tube was placed in a graphite oven and pyrolyzed for 1 h at 100 °C with the lid, 2 h at 150 °C without the lid, 1 h at 170 °C without the lid, and 10 min at 170 °C with the lid. After pyrolysis, a colorless and transparent solution was obtained. The solution was transferred to a 50-mL volumetric flask with 1% nitric acid, and the volume of the solution was adjusted to 50 mL with 1% nitric acid. Finally, 0.5 mL of the solution was diluted to 1 L with 1% nitric acid, prior to analysis in the ICP-OES. The solutions used for the standard curves ranged from 0 to 10 mg/L of NaCl. A standard solution of NaCl was prepared from a 1 mg/mL stock solution in 1% hydrochloric acid (Merck, Kenilworth, NJ, USA).

#### 2.5.4. Conductimetry

The conductivity experiments were carried out using two different devices. The NaCl content was measured using a digital conductivity meter (DDS-307A, INESA Scientific Instrument Co., Shanghai, China) equipped with a DJS-1C probe (INESA Scientific Instrument Co., Shanghai, China). The microparticles were dispersed in deionized water, and crushed prior to titration. The NaCl cumulative release in deionized water was monitored with a T50 titrator equipped with an Inlab 730 probe and an internal agitation system (Mettler Toldeo, Shanghai, China). The microparticles were loaded into the conductimeter cell containing 80 mL of deionized water. The release of NaCl was monitored over 20 min under agitation. All of the measurements were performed in triplicate.

### 2.6. Preparation of Glucose–Lysine–Microparticle Model Systems

A model system consisting of lysine, glucose, and NaCl microparticles was used to assess the efficacy of the encapsulation in modulating pyrraline formation. The model systems were prepared, as previously described for the glucose–lysine–NaCl model systems, with a few modification. The details of all of the formulations are shown in Table 1. Briefly, 0.1 mmol lysine, 0.1 mmol glucose, and NaCl microparticles (set concentration) were transferred to a 25-mL PTFE tube. The total reaction volume was adjusted to 15 mL using deionized water. The final concentration of Na^+^ (not NaCl) in the reaction solution was set to 0.50%. The thermal treatments were performed in an oil bath at 140 °C for 20 min. All of the reactions were performed in triplicate. The following procedures of derivatization were performed according to the previous procedures in Section 2.2.

### 2.7. Extent of Browning

The extent of browning in the model systems was assessed with a TU-1810 UV/VIS spectrophotometer (Beijing Puxi Instrument Co., Beijing, China) at 420 nm, following appropriate dilutions. The color intensities of the samples were presented as absorbance values after multiplication with the dilution factor.

### 2.8. Pyrraline and 3-DG Analysis

#### 2.8.1. Solid Phase Extraction (SPE) Procedures

The sample purification was performed by the SPE procedures, according to our previous study [27].

#### 2.8.2. LC-MS Analysis

The pyrraline and 3-DG were analyzed by LC-MS, according to our previous study, with some modifications [27]. Briefly, pyrraline was quantified by its molecular ion peak (*m*/*z* = 255.13), and 3-DG was determined by the molecular ion peak of 3-DG_qx_ (*m*/*z* = 235.10). The monitoring time was 15 min. An external calibration was performed with standards.

### 2.9. Statistical Analysis

The statistically significant differences among the means from the triplicate analyses (*p* < 0.05) were determined by Duncan’s multiple range test using SPSS (version 12.0 for Windows, SPSS Inc., Chicago, IL, USA).

## 3. Results and Discussion

### 3.1. Effect of NaCl in Glucose–Lysine–NaCl Model Systems

#### 3.1.1. Effect of NaCl on the Browning Intensity

**Figure 1:**
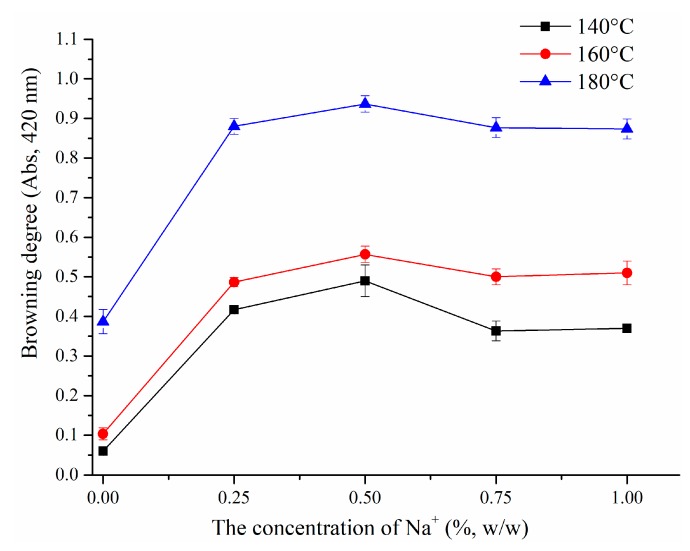
Browning intensity (λ = 420 nm) at different sodium concentrations in model reaction systems.

Figure S1 (see Supplementary Materials) shows the browning development with 0.00% to 1.00% Na^+^ at 140–180 °C in glucose–lysine–NaCl model systems. Figure S2 (see Supplementary Materials) shows the browning development with heating time. The findings revealed that with the increasing temperature and heating time, the browning intensity increased. This conclusion is partially confirmed by the absorbance results at 420 nm (Figure 1). MR has a relatively high temperature coefficient (Q_10_ values range between 2 and 8) [34]; therefore, the heating temperatures can significantly accelerate the MR.

However, an interesting trend occurred in the browning intensity when we investigated the effect of NaCl. Figure S1 (see Supplementary Materials) shows that the browning development does not accelerate continuously with an increase in Na^+^ content. The curves in Figure 1 show that the maximum browning intensity occurred at 0.50% Na^+^ in all of the heating treatments. At 140, 160, and 180 °C, the color intensity of the MR products was 8.2, 5.6, and 2.4 times higher, respectively, at 0.50% Na^+^ than at 0.00% Na^+^. In addition, browning intensity increased significantly from 0.00 to 0.25% Na^+^ and increased slowly from 0.25 to 0.50% Na^+^. At > 0.50% Na^+^, the browning intensity gradually decreased. These results were partially consistent with the results reported by Kwak [35]. The color intensity of the MR products was enhanced by ~5% with 1% NaCl (i.e., 0.4% Na^+^) and inhibited by 20% with 10% NaCl (i.e., 4% Na^+^). However, in this study, the 0.25–1.00% Na^+^ enhanced the browning of the MR products heated at 140 to 180 °C for 20 min compared with 0.00% Na^+^. The main reason for this difference may be the low Na^+^ and the absence of the phosphate buffer in our study. Compared to the study by Kwak, our study used high temperature and short time heating treatments.

The effect of Na^+^ on the Maillard browning appears to be quite intricate, because there are contradictory reports in the literature. A high NaCl increased the browning in the cereal model systems (Na^+^ at 0% to 2.14%) [36] and in the breakfast cereals (Na^+^ at 0% to 2.18%) [37]. Moreau el al. reported that the browning intensity promoted by NaCl is neither related to the NaCl hygroscopic behavior, nor to the physical state of the model systems (glassy vs rubbery) [36]. In addition, Rizzi observed that NaCl (0.04 M, ~ mass % of Na^+^ at 0.1%) increased the Maillard browning in aqueous pH 7.2-buffered (bis/tris) solutions of a ribose–glycine system [38]. However, Kwak and Lim concluded that the Maillard browning can be greatly inhibited by the high NaCl concentrations (e.g., 1% or 10%; mass percent of Na^+^ at either 0.4% or 4%) in aqueous binary mixtures of glucose and different amino acids in a pH 6.5 citrate–phosphate buffer heated at 100 °C for 6 h [35]. Similarly, a 5% or 10% NaCl (mass % of Na^+^ at 2% or 4%) can weaken the Maillard browning in aqueous binary mixtures of glucose, and in different amino acids in a citrate or phosphate buffer heated at 100 °C after 60 min [39]. These differences may be attributed to the role of the phosphate buffer solutions in the mixtures. The phosphate anion enhances the Maillard browning by providing reactive intermediates directly from sugars [40,41].

With respect to the enhancement of the Maillard browning by NaCl, the evidence suggests that the NaCl addition promotes the interconversion of glucose–fructose and the accumulation of 1-deoxyglucosone (1-DG) and 3-DG [42], which can increase the browning intensity during the final stage of MR. This evidence will be further discussed in the section on 3-DG formation.

#### 3.1.2. Effect of NaCl on Pyrraline Formation

**Figure 2:**
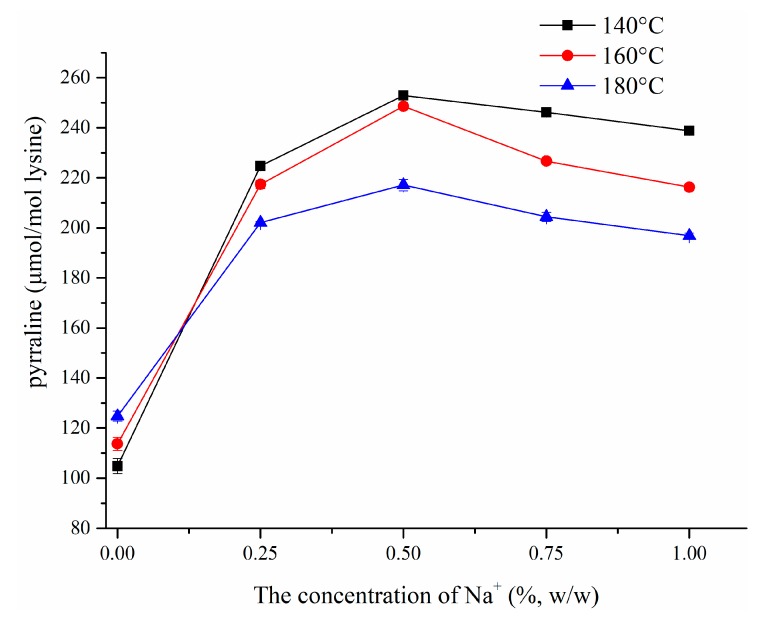
Sodium and temperature-dependent formation of pyrraline in model reaction systems.

Figure 2 shows the pyrraline concentrations at different Na^+^ levels. The maximum pyrraline concentrations (252.90 ± 0.36 μmol/mol lysine at 140 °C, 248.63 ± 0.66 μmol/mol lysine at 160 °C, and 217.13 ± 2.31 μmol/mol lysine at 180 °C) were obtained at 0.50% Na^+^. At 0.00–0.25% Na^+^, the pyrraline content increased rapidly (104.81 ± 3.03 to 224.76 ± 1.55 μmol/mol lysine at 140 °C, 113.68 ± 2.65 to 217.38 ± 1.72 μmol/mol lysine at 160 °C, and 124.79 ± 2.02 to 202.15 ± 0.39 μmol/mol lysine at 180 °C) in each temperature group. The pyrraline concentration continued to increase at 0.25–0.50% Na^+^. However, at >0.50% Na^+^, the pyrraline concentration decreased gradually. In this study, the maximum pyrraline concentration was obtained at 140 °C, consistent with our past findings [27,28].

The results obtained from acrylamide, a food contaminant derived from MR, were different to the pyrraline results. Levine and Ryan found that the acrylamide formation in a dough model with 0.02 M NaCl (mass % of Na^+^ at ~0.02% based on the dough weight) did not differ from the control (0% Na^+^), while 0.04 M (~0.04% Na^+^) and 0.08 M (~0.07% Na^+^) NaCl significantly reduced the acrylamide content by ~23% [21]. Unexpectedly, the increase in NaCl from 0.08 M (~0.07% Na^+^) to 0.44 M (~0.40% Na^+^) had insignificant effects on the acrylamide formation. However, some studies have reported that the acrylamide formation can be reduced by 1% to 2% NaCl (0.4 to 0.8% Na^+^) in bread and roll production at 220 °C for 30 min [22], and by 1.3% NaCl (0.52% Na^+^) in a cracker model baked at 180 °C for 15 min [43]. The differing effects of NaCl on pyrraline and acrylamide formation can be attributed to the following three aspects. First, acrylamide formation occurs at relatively high temperatures (up to 220 °C) with dry heat treatment, whereas pyrraline was produced at 120–180 °C with humid heat in our study. Second, acrylamide is formed predominantly by sugars reacting with asparagine, while pyrraline is mainly formed from sugars reacting with lysine. Third, the dough and cracker models [21,43] are more complex than the simplified models used in our study.

In MR, pyrraline is formed from 3-DG between a reducing sugar and the epsilon-amino group of lysine [44]. The effect of NaCl on pyrraline formation may be linked to changes in the pyrraline formation pathway. Hodge [45] classified MR into the following three stages: initial, intermediate, and final stages. During the initial stage, sugar–amine condensation and Amadori rearrangement occur, resulting in Amadori rearrangement products without absorption in the ultraviolet region. During the intermediate stage, complex reactions involving sugar dehydration, fragmentation, and amino acid degradation (Strecker degradation) occur, with a strong absorption in the ultraviolet region. During the final stage, highly colored products are formed via aldol condensation, aldehyde–amine condensation, and heterocyclic nitrogen compound synthesis. Therefore, it is possible that NaCl alters these complex reactions.

Sugar dehydration and fragmentation are affected by Na^+^; this will be discussed in the following section. The isomerization of glucose into fructose, which can occur in MR [28], facilitates pyrraline formation, because of the high reactivity of fructose [27]. In addition, NaCl may promote a constant isomerization rate [46]. The interaction of NaCl with glucose catalyzes the mutarotation and isomerization of glucose into fructose [47]. The sugar–metal coordination is responsible for the reaction; the metal interacts with the hemiacetal portion of glucopyranose [47]. Na^+^ may increase the rate constants, especially with the transition states in a particular stereochemistry, of the dehydration of the intact fructofuranose ring [48].

#### 3.1.3. Effect of NaCl on 3-DG Formation

**Figure 3:**
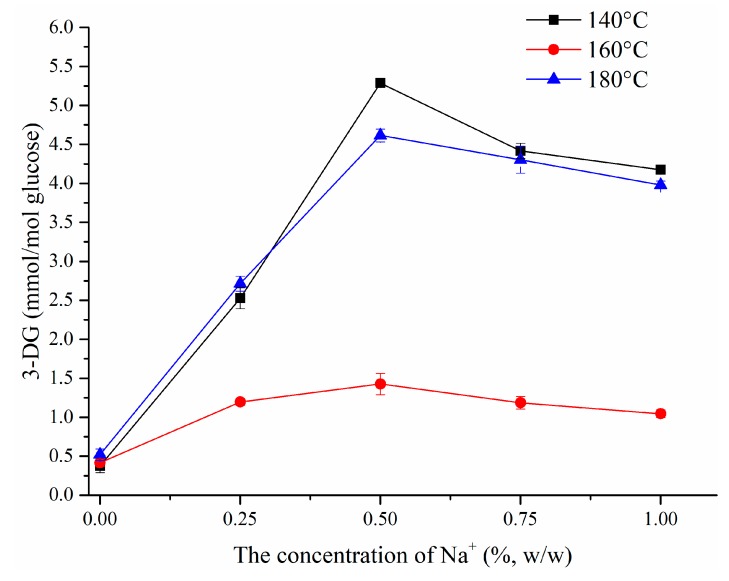
Sodium and temperature-dependent formation of 3-DG in model reaction systems.

Figure 3 shows that the 3-DG concentration increased (0.38 ± 0.09 to 5.29 ± 0.04 mmol/mol glucose at 140 °C and 0.52 ± 0.07 to 4.61 ± 0.08 mmol/mol glucose at 180 °C) from 0.00% to 0.50% Na^+^. However, a slight rise in 3-DG concentration (0.42 ± 0.05 to 1.43 ± 0.14 mmol/mol glucose) was observed, from 0.00% to 0.50% Na^+^ at 160 °C. At >0.50% Na^+^, the 3-DG concentration gradually decreased in each temperature group. In general, the changes in the 3-DG (Figure 3) were consistent with the changes in pyrraline (Figure 2) at the same temperature, except for at 160 °C. The exception can be because some other nitrogen compounds derived from 3-DG may be produced easier than pyrraline at 160 °C [49].

3-DG is the key intermediate in pyrraline formation; therefore, NaCl may affect pyrraline formation by modifying the sugar dehydration and fragmentation. As aforementioned, sugar dehydration and fragmentation mainly occur during the intermediate stage of MR. In this stage, Na^+^ forms complexes with carbohydrate or intermediates to stabilize a particular conformation that facilitates ring opening during the process of dehydration and fragmentation [50]. Furthermore, computational evidence suggests that Na^+^ directly interacts with nucleophilic oxygen molecules [51], which are central during carbohydrate pyrolysis, dehydration, and isomerization [48]. In fact, 1, 2-dehydration reactions of open D-glucose to dicarbonyl compounds, and 1, 2-dehydration reactions of β-D-glucose to cyclic enols may be catalyzed in the presence of Na^+^ [48]. The former is involved in 3-DG formation and exerts a powerful effect on the pyrraline formation pathway, and the latter contributes to 5-hydroxymethylfurfural (5-HMF) formation. Consequently, these computational results may be partly responsible for the acceleration of 3-DG formation with NaCl. In this study, 3-DG gradually decreased at Na^+^ >0.50% (Figure 3). This result suggests that the 1, 2-dehydration reactions of β-D-glucose to cyclic enols may be strengthened, whereas the 1, 2-dehydration reactions of open D-glucose to dicarbonyl compounds may be weakened.

However, the evidence has shown that the formation of 3-DG, which is the dominant component of α-dicarbonyl compounds, decreased in the presence of NaCl (~ mass % of Na^+^ at 0.23%) [46]. The presence of 1.5% NaCl (~0.6% Na^+^) had no effect on the formation of α-dicarbonyl compounds [42]. These inconsistent results may be attributed to the dominant role of 1, 2-dehydration reactions of β-D-glucose into cyclic enols during sugar dehydration and fragmentation.

In summary, the results revealed that NaCl plays a dual role in 3-DG formation during sugar dehydration and fragmentation. A low Na^+^ (<0.50%) facilitates 3-DG formation in MR, whereas a high Na^+^ (>0.50%) impedes 3-DG formation. It has been proposed that Na^+^ changes the reaction rate coefficients of glucose pyrolysis to varying degrees, with approximately 70% of the reactions being promoted by Na^+^, approximately 25% of the reactions being inhibited by Na^+^, and the rest not being affected [48]. Moreover, the effect of salt on the rate constants of α-dicarbonyl compound formation varies with the precursor, compounds, and temperature [46].

### 3.2. NaCl Encapsulation Modulates Pyrraline Formation

Past studies on NaCl encapsulation assessed the release of oil electrolytes (e.g., canola oil and triglycerides) in water emulsion systems [52,53]. Lipid-coated NaCl reduced the 5-HMF and acrylamide formation in MR [23]. However, the use of NaCl encapsulation to modulate AGEs formation has never evaluated.

NaCl can be successfully encapsulated using spray coating. Microparticles with mean diameters of 789, 866, 773, 766, 780, and 795 μm were obtained for NaCl coated with WMS, NMS, HAMS, GRS, XG, and GA, respectively (Table 2). The NaCl content was measured by the conductimetry and titrated at 284 ± 12, 269 ± 6, 258 ± 8, 247 ± 10, 273 ± 16, and 288 ± 15 mg/g of microparticles (coated with WMS, NMS, HAMS, GRS, XG, and GA, respectively). The NaCl content in the microparticles provided an important basis for the formulation design (Table 1).

#### 3.2.1. Thermal (DSC) Properties of NaCl Microparticles

The DSC thermograms of the NaCl microparticles are shown in Figure S3–S8 (see Supplementary Materials). For the starch-based coating materials, the peak in the DSC thermograms can be attributed to the dissociation in the double helix of amylopectin [54]. XG is a polysaccharide with a linear and branched chain structure [55], and GA is a mixture of glycoproteins and polysaccharides with branched chains [56]. Therefore, gums with structures similar to starch-based coating materials may result in the peak in the DSC thermograms of XG and GA.

The transition temperatures, onset (*T_o_*), peak (*T_p_*), conclusion (*T_c_*), and enthalpy change (*ΔH*) are presented in Table 3. In the starch- and gum-coated microparticles, the transition temperatures had the following order: GA-coated NaCl ≈ WMS-coated NaCl < NMS-coated NaCl < HAMS-coated NaCl < GRS-coated NaCl ≈ XG-coated NaCl. In the starch-coated microparticles, this result is caused by the content of amylose and amylopectin. A high amylose content and low amylopectin content can result in high transition temperatures of starch. In the gum-coated microparticles, the structure and content of the polysaccharides in the gum may lead to different transition temperatures. It can be hypothesized that the coated NaCl microparticles with a high transition temperature may exhibit a high heat resistance, thereby allowing for a slow release rate of NaCl.

#### 3.2.2. Morphology of NaCl Microparticles

**Figure 4:**
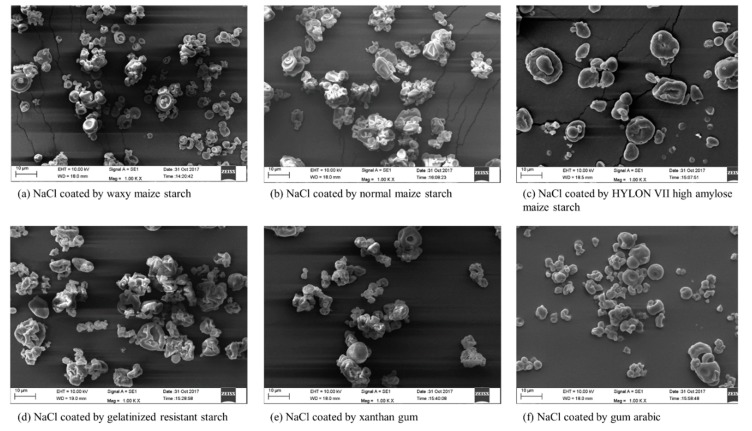
NaCl microparticles obtained from SEM.

The shape and surface morphology of the NaCl microparticles were obtained by SEM. Figure 4 shows that the starch-coated NaCl microparticles exhibit the morphology of maize starch. All of the NaCl microparticles had smooth surfaces. In addition, we did not obtain any exposed NaCl crystals.

From the perspective of physical chemistry, a more homogeneous structure and smooth surface of the coating material can enhance the barrier properties [57]. Therefore, it can be inferred that a more homogeneous structure and smooth surface of NaCl microparticles can contribute to a low release of NaCl from the microparticles in the solution. Water or other solvents can access the microparticle with a heterogeneous structure and roughness surface, resulting in a fast release of NaCl.

#### 3.2.3. NaCl Release from Microparticles

**Figure 5:**
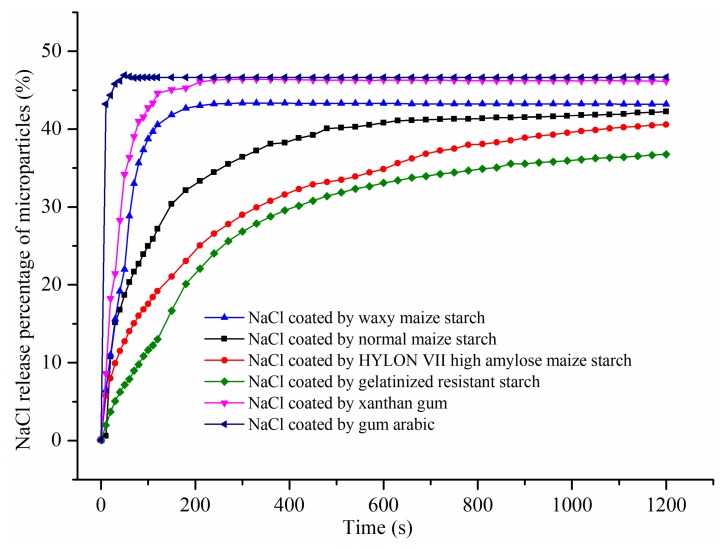
NaCl cumulative release profiles in deionized water.

The barrier ability of the coating material to isolate NaCl from water was measured by the conductimetry. The release of NaCl was monitored over time by plotting the increase of water conductivity after the dispersion of microparticles in water (Figure 5). Compared with deionized water, no significant fluctuations in water conductivity were obtained with blank microparticles made of the coating material (data not shown). This result suggests that the coating materials do not lead to a NaCl release in the aqueous solution. In addition, the release rates of NaCl from the microparticles decreased in the following order: GA-coated NaCl > XG-coated NaCl > WMS-coated NaCl > NMS-coated NaCl > HAMS-coated NaCl > GRS-coated NaCl. It can be proposed that a slow NaCl release from the microparticles may delay the participation of NaCl in MR, thereby affecting the 3-DG and pyrraline formation.

However, the results of the NaCl release were not consistent with the transition temperatures obtained from DSC. In general, a high release rate of NaCl can be observed from starch-coated microparticles with low transition temperatures. However, the XG-coated NaCl with high transition temperatures had a high release rate of NaCl. The transition temperatures of the GA-coated NaCl were approximately equal to those of the WMS-coated NaCl, whereas the former had a higher release rate of NaCl compared with the latter. The inconsistency of the gum-coated microparticles may be caused by their high solubility in an aqueous solution. GA and XG can readily dissolve in water at low temperatures (below 60 °C) [55,56], while starches usually dissolve in water after gelatinization with heat treatment [58]. The high solubility of GA and XG can lead to a rapid disintegration of the the coating material and a rapid release of NaCl from the gum-coated microparticles.

#### 3.2.4. Browning Extent and Pyrraline Formation with the Addition of NaCl Microparticles

**Figure 6:**
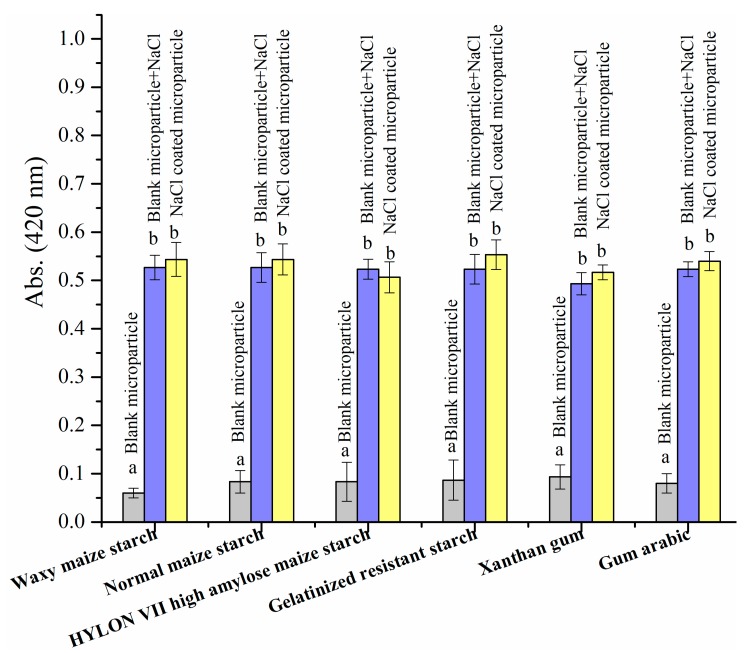
Extent of browning (absorbance at 420 nm) in the model systems prepared with different formulations (refer to Table 1 for the formulations). Values with the same letter within a column are not significantly different (*p* > 0.05, Duncan’s test).

The effect of NaCl encapsulation on the Maillard browning is presented in Figure 6. NaCl mixed with blank microparticles and NaCl-coated microparticles significantly accelerated the extent of the browning (~five times as high as that without NaCl). These results were consistent with the results presented in Figure 3. The findings confirmed that MR can be accelerated by NaCl. However, there was no significant difference in the extent of browning between NaCl mixed with blank microparticles and NaCl-coated microparticles. In addition, no significant difference in the extent of the browning was obtained among the groups. This finding was unexpected, and suggests that NaCl encapsulation has no impact on the browning, at least after the completion of MR. Furthermore, the findings indicate that NaCl is fully released from the microparticles following heat treatment.

**Figure 7:**
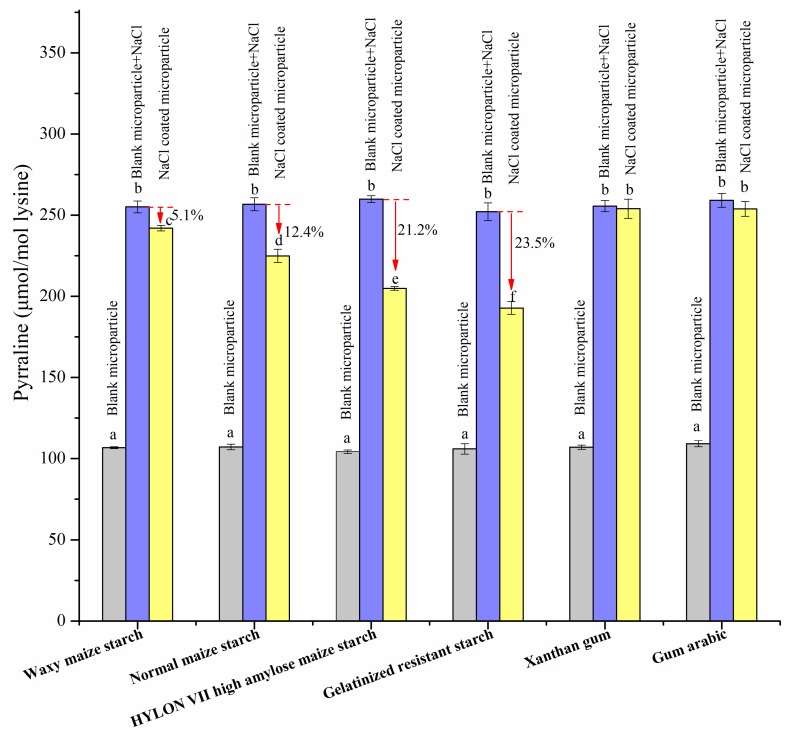
Concentration of pyrraline (μmol/mol lysine) in model systems prepared with different formulations (refer to Table 1 for the formulations). Values with the same letter within a column are not significantly different (*p* > 0.05, Duncan’s test).

Figure 7 shows the pyrraline formation in different model systems. In each group, NaCl significantly promoted pyrraline formation. However, starch-coated NaCl retarded the pyrraline formation when compared to NaCl mixed with blank microparticles. The inhibition of pyrraline formation by NaCl-coated microparticles exhibited the following order: WMS-coated NaCl (5.1%) < NMS-coated NaCl (12.4%) < HAMS-coated NaCl (21.2%) < GRS-coated NaCl (23.5%). Unexpectedly, in the gum-coated NaCl, we obtained no significant differences in the pyrraline concentration between the NaCl mixed with blank microparticles and the NaCl-coated microparticles. These findings indicate that starch-coated microparticles with high transition temperatures reduce pyrraline formation, whereas gum-coated microparticles have little effect. The high transition temperatures of starch can be attributed to the high amylose and low amylopectin contents. These molecules with a high heat resistance may delay the NaCl release to the reaction solution, thereby affecting the pyrraline formation. Even though XG has a high heat resistance and GA has a heat resistance close to WMS, their high solubility weakens their barrier ability between NaCl and the reaction solution. In summary, the delay of NaCl release to the reaction solution can reduce the pyrraline formation in MR, with little effects on the browning.

#### 3.2.5. 3-DG Formation with the Addition of NaCl Microparticles

**Figure 8:**
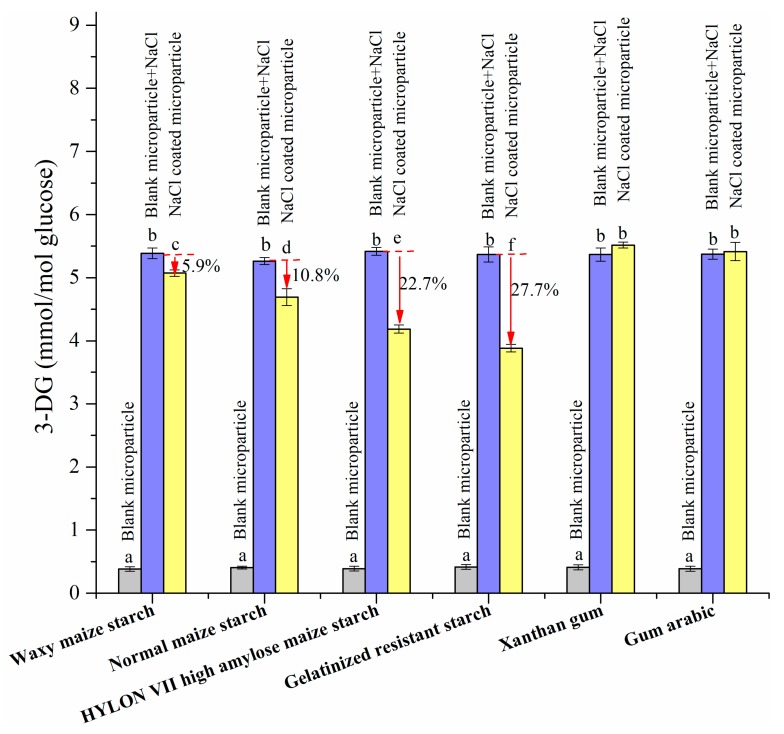
Concentration of 3-DG (mmol/mol glucose) in model systems prepared with different formulations (refer to Table 1 for the formulations). Values with the same letter within a column are not significantly different (*p* > 0.05, Duncan’s test).

The key intermediate of pyrraline formation, 3-DG, was monitored so as to better understand the effect of NaCl encapsulation on pyrraline formation in MR. Figure 8 shows that NaCl mixed with blank microparticles and NaCl-coated microparticles significantly promoted 3-DG formation. The blockage of NaCl-coated microparticles on 3-DG formation can be compared in the following order: WMS-coated NaCl (5.9%) < NMS-coated NaCl (10.8%) < HAMS-coated NaCl (22.7%) < GRS-coated NaCl (27.7%). This trend is similar to the trend obtained in pyrraline. These results indicate that starch-coated NaCl microparticles with a high heat resistance may weaken the 3-DG formation, because of the delay of NaCl release to the reaction solution. Moreover, similar to the results of pyrraline, the gum-coated NaCl microparticles with a high solubility limit the decrease in 3-DG formation.

Consequently, it can be proposed that the pyrraline formation prevented by the NaCl encapsulation stems from the effect of NaCl encapsulation via 3-DG formation. As previously mentioned, in the intermediate stage of MR, the 1, 2-dehydration reactions of open D-glucose to dicarbonyl compounds, and the 1, 2-dehydration reactions of β-D-glucose to cyclic enols can be catalyzed in the presence of Na^+^ [48]. The former plays a major role in 3-DG formation. Therefore, the delay in the NaCl release may modulate the 1, 2-dehydration reactions of glucose to dicarbonyl compounds and cyclic enols, thereby affecting 3-DG formation. Future studies should investigate this proposed mechanism.

## 4. Conclusions

In MR, NaCl significantly increased the browning intensity and pyrraline formation, with a rapid increase in 3-DG concentration. NaCl encapsulation is an effective strategy to mitigate pyrraline formation. NaCl-coated microparticles with a high heat resistance may prevent pyrraline via 3-DG, whereas the high solubility of the coating material offsets the effect of NaCl encapsulation. However, NaCl encapsulation has little impact on the browning intensity. The transition temperature and solubility of the coating material are the key factors that affect pyrraline formation. Encapsulation may delay the NaCl participation in MR, particularly during the initial and intermediate stages.

## Figures and Tables

**Table 1:** The formulations used to prepare the model systems in a PTFE-lined tube ^a.^

Setup	Amount in the PTFE Lined Tube (mg)		
	NaCl	Coated NaCl Microparticles	Coating Materials Microparticles
NaCl 0.000% (Na^+^ 0.00%)	0		
NaCl 0.625% (Na^+^ 0.25%)	94		
NaCl 1.250% (Na^+^ 0.50%)	188		
NaCl 1.875% (Na^+^ 0.75%)	281		
NaCl 2.500% (Na^+^ 1.00%)	375		
encapsulated NaCl (WMS)		662	
blank microparticle (WMS) + NaCl	188		474
blank microparticle (WMS)			474
encapsulated NaCl (NMS)		699	
blank microparticle (NMS) + NaCl	188		511
blank microparticle (NMS)			511
encapsulated NaCl (HAMS)		729	
blank microparticle (HAMS) + NaCl	188		541
blank microparticle (HAMS)			541
encapsulated NaCl (GRS)		761	
blank microparticle (GRS) + NaCl	188		573
blank microparticle (GRS)			573
encapsulated NaCl (XG)		689	
blank microparticle (XG) + NaCl	188		501
blank microparticle (XG)			501
encapsulated NaCl (GA)		653	
blank microparticle (GA) + NaCl	188		465
blank microparticle (GA)			465

^a^ The following components were common for all of the formulations: glucose, 0.018 g; lysine, 0.015 g; and deionized water, 15 g. The data of the NaCl and coating materials were based on the results of Section 2.5.3. PTFE—polytetrafluoroethylene; WMS—waxy maize starch; NMS—normal maize starch; HAMS—HYLON VII high amylose maize starch; GRS—gelatinized resistant starch; XG—xanthan gum; GA—gum arabic.

**Table 2:** Particle size distribution of NaCl-coated microparticles (sieving mesh sizes no. 100) ^a^.

Sample	D_3,2_ (μm)
NaCl coated by WMS	789
NaCl coated by NMS	866
NaCl coated by HAMS	773
NaCl coated by GRS	766
NaCl coated by XG	780
NaCl coated by GA	795

^a^ WMS—waxy maize starch; NMS—normal maize starch; HAMS—HYLON VII high amylose maize starch; GRS—gelatinized resistant starch; XG—xanthan gum; GA—gum arabic.

**Table 3:** Thermal properties of NaCl-coated microparticles treated with heat moisture and annealing ^a^.

Samples	Gelatinization of Coating Materials				
	*T_o_* (°C)	*T_p_* (°C)	*T_c_* (°C)	*ΔH* (J/g)	*T_c_* -*T_o_* (°C)
NaCl coated by WMS	55.79	58.26	63.08	2.770	7.29
NaCl coated by NMS	93.42	102.37	113.28	2.133	19.86
NaCl coated by HAMS	130.12	130.71	132.70	0.212	2.58
NaCl coated by GRS	155.55	158.65	161.53	0.539	5.98
NaCl coated by XG	150.31	157.55	163.61	3.305	13.30
NaCl coated by GA	57.20	57.90	60.97	1.301	3.77

^a^ WMS—waxy maize starch; NMS—normal maize starch; HAMS—HYLON VII high amylose maize starch; GRS—gelatinized resistant starch; XG—xanthan gum; GA—gum arabic.

## Supplementary Materials

The following are available online at https://www.mdpi.com/2218-273X/9/11/721/s1: Figure S1: Browning development with 0.00 to 1.00% sodium in glucose-lysine-NaCl model systems; Figure S2: Browning development from 5 to 20 min in glucose-lysine-NaCl model systems; Figure S3: Thermograms (DSC) of NaCl encapsulated microparticles (waxy maize starch coating); Figure S4: Thermograms (DSC) of NaCl encapsulated microparticles (normal maize starch coating); Figure S5: Thermograms (DSC) of NaCl encapsulated microparticles (HYLON VII high amylose maize starch coating coating); Figure S6: Thermograms (DSC) of NaCl encapsulated microparticles (gelatinized resistant starch starch coating); Figure S7: Thermograms (DSC) of NaCl encapsulated microparticles (xanthan gum coating); Figure S8: Thermograms (DSC) of NaCl encapsulated microparticles (gum arabic coating).

## Abbreviations

AGEs—advanced glycation end products; d-AGEs—dietary AGEs; MR—Maillard reaction; CML—N^ε^-carboxymethyllysine; CEL—N^ε^-carboxyethyllysine; HMF—hydroxymethylfurfural; 1-DG—1-deoxyglucosone; 3-DG—3-deoxyglucosone; WMS—waxy maize starch; NMS—normal maize starch; HAMS—HYLON VII high amylose maize starch; GRS—gelatinized resistant starch; XG—xanthan gum; GA—gum arabic.
