# Supplementary material for: Pyrraline Formation Modulated by Sodium Chloride and Controlled by Encapsulation with Different Coating Materials in the Maillard Reaction
Source: Biomolecules. 2019 Nov 10;9(11):721. doi: 10.3390/biom9110721 (PMC6920989; doi:10.3390/biom9110721)
Supplement: Supplementary file 1 [file biomolecules-09-00721-s001.pdf]

Supplementary Materials: Pyrraline Formation Modulated by Sodium Chloride and Controlled by Encapsulation with Different Coating Materials in Maillard Reaction

Zhili Liang [1,*], Xu Chen [2], Zhao Yang [1],Yuzhu Lai [1], Yinling Yang [1], Chuying Lei [1],Ya Zeng [1]

[1] School of Food Science, Guangdong Food and Drug Vocational College, Guangzhou 510520, China
[2] Engineering Research Center of Health Food Design & Nutrition Regulation, School of Chemical Engineering and Energy Technology, Dongguan University of Technology, Dongguan 523808, China

Corresponding author
[*] Zhili Liang, Tel./ Fax: +86-20-29164643, Email address: zhililiang1988@gmail.com/ liangzl@gdyzy.edu.cn

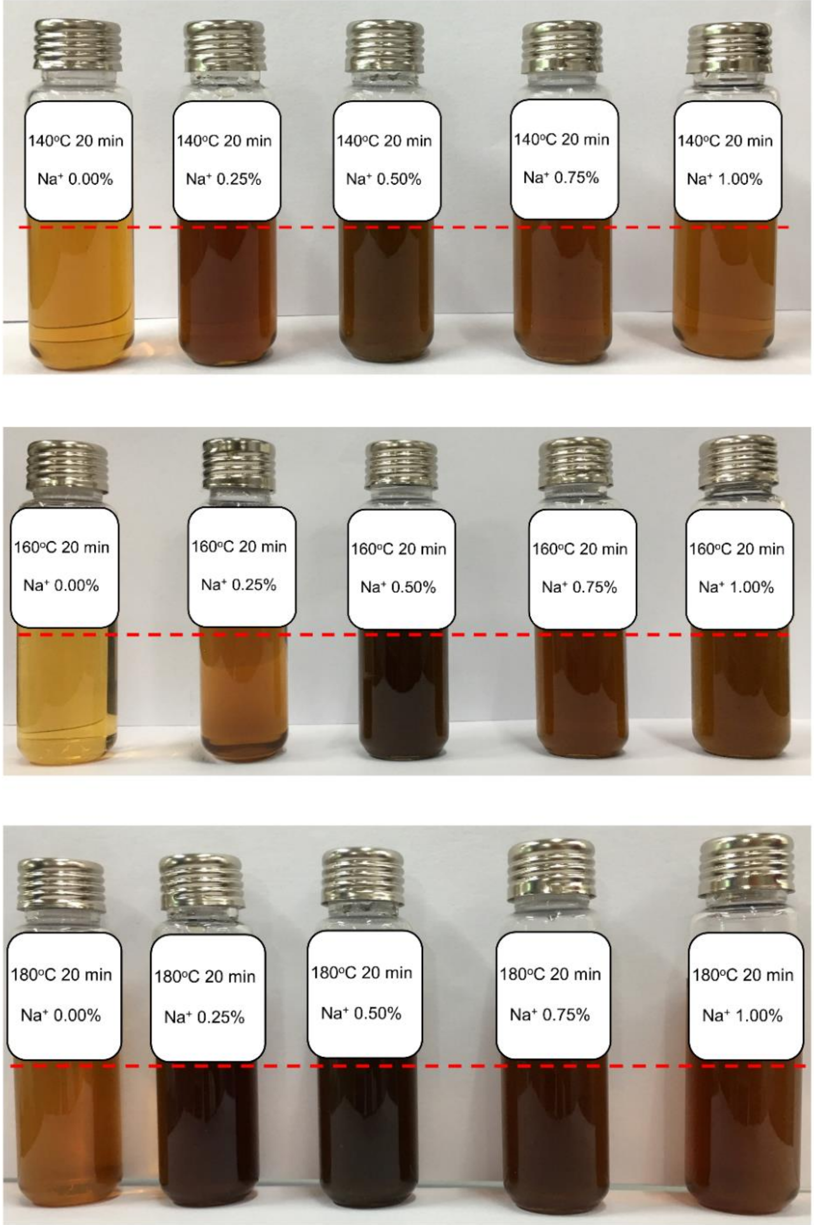

Figure S1. Browning development with 0.00 to 1.00% sodium in glucose-lysine-NaCl model systems

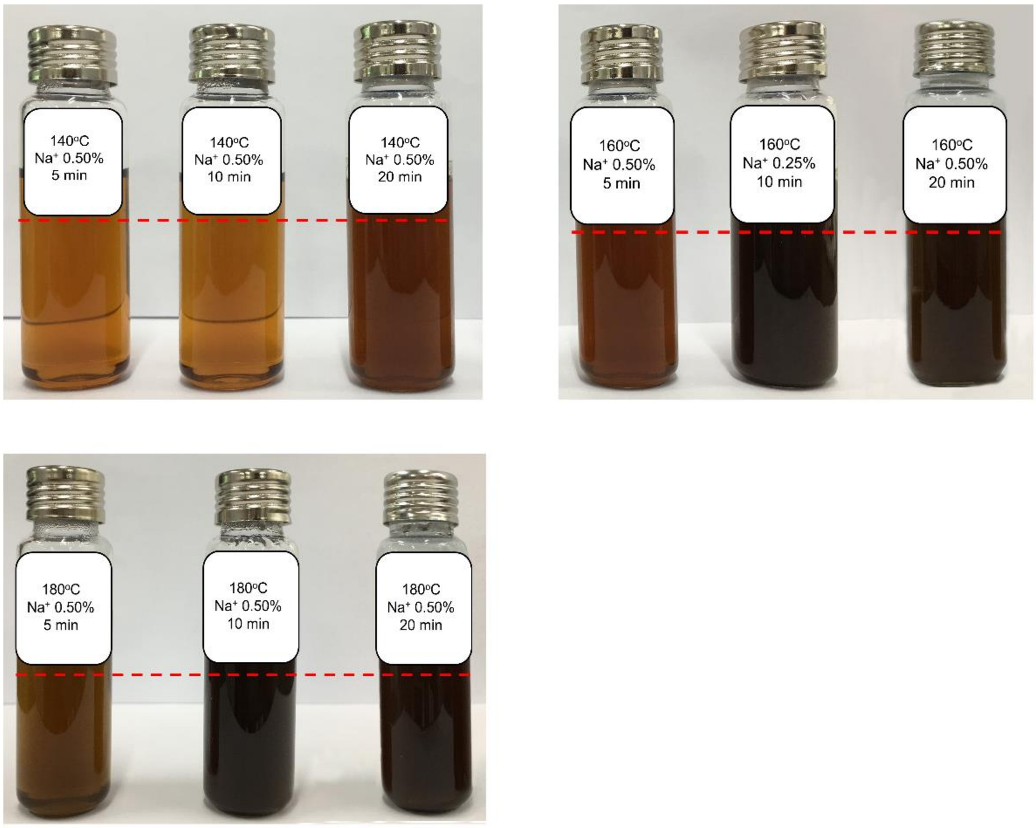

Figure S2. Browning development from 5 to 20 min in glucose-lysine-NaCl model systems

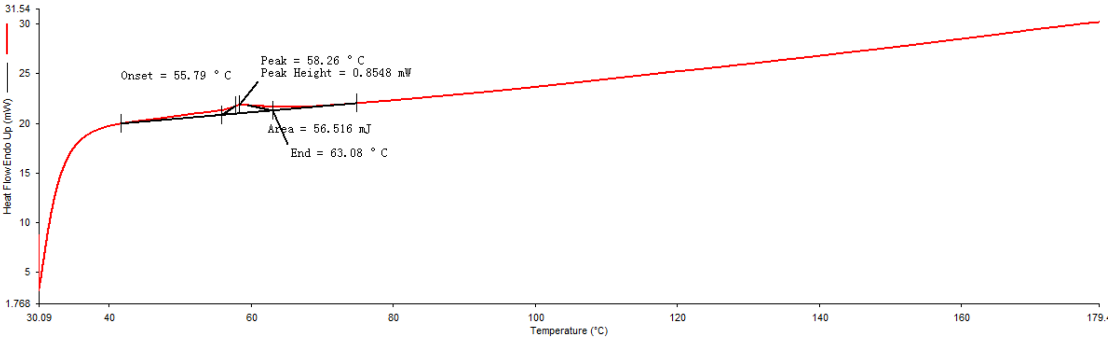

Figure S3. Thermograms (DSC) of NaCl encapsulated microparticles (waxy maize starch coating)

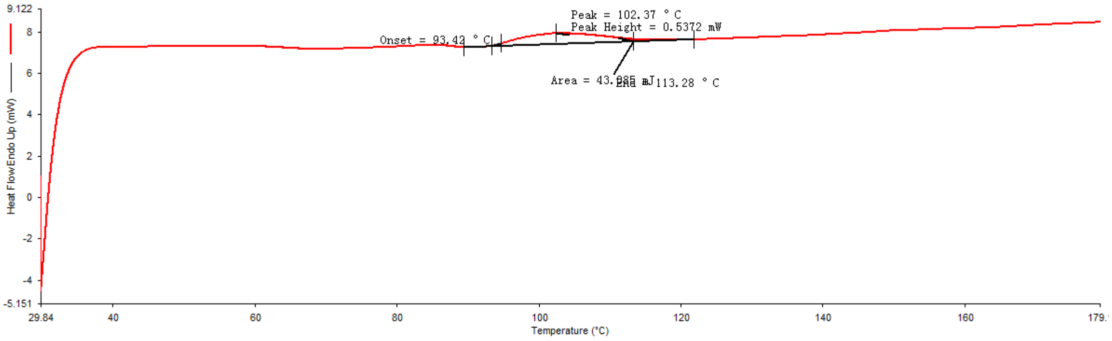

Figure S4. Thermograms (DSC) of NaCl encapsulated microparticles (normal maize starch coating)

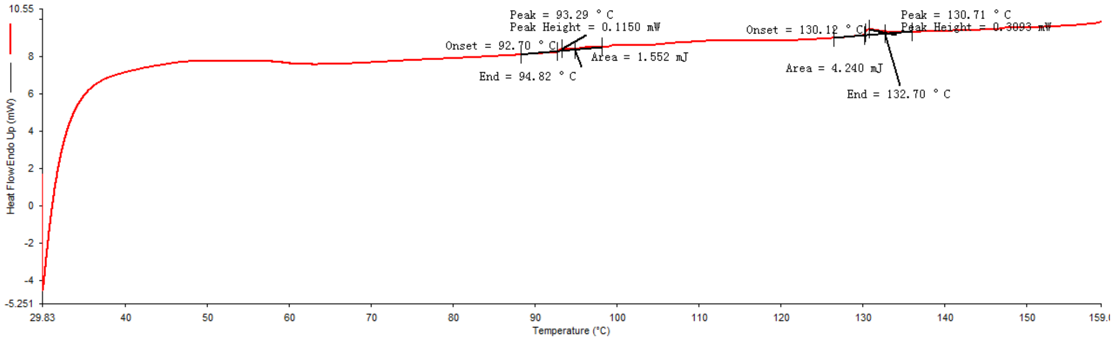

Figure S5. Thermograms (DSC) of NaCl encapsulated microparticles (HYLON VII high amylose maize starch coating coating)

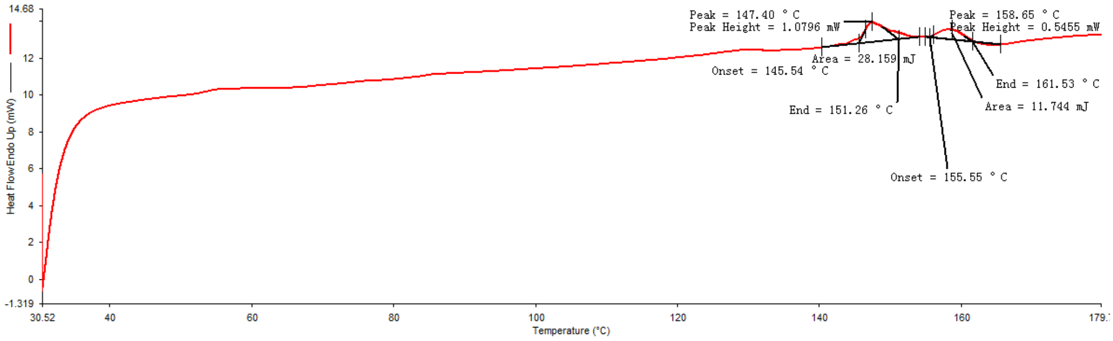

Figure S6. Thermograms (DSC) of NaCl encapsulated microparticles (gelatinized resistant starch starch coating)

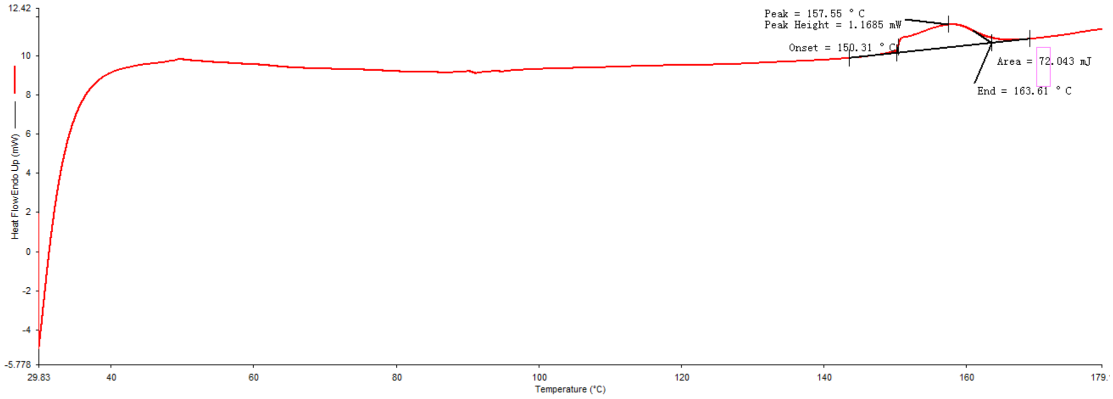

Figure S7. Thermograms (DSC) of NaCl encapsulated microparticles (xanthan gum coating)

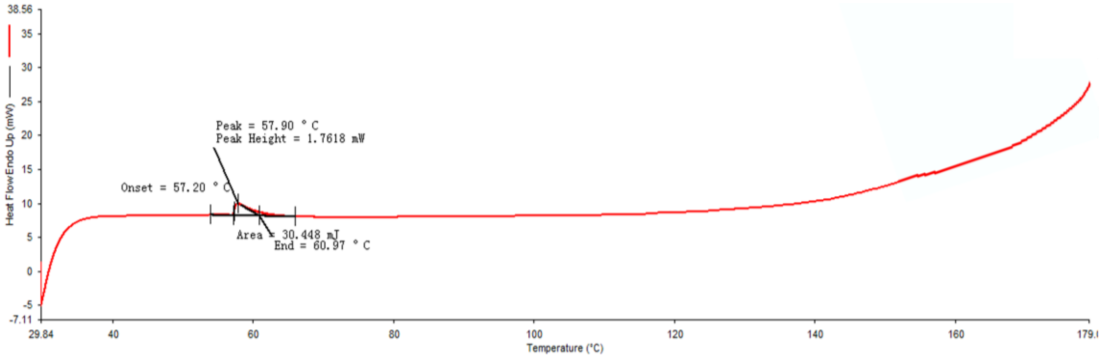

Figure S8. Thermograms (DSC) of NaCl encapsulated microparticles (gum arabic coating)
